# Multi-Fiber Tractography Visualizations for Diffusion MRI Data

**DOI:** 10.1371/journal.pone.0081453

**Published:** 2013-11-25

**Authors:** Sjoerd B. Vos, Max A. Viergever, Alexander Leemans

**Affiliations:** Image Sciences Institute, University Medical Center Utrecht, Utrecht, The Netherlands; Beijing Normal University, China

## Abstract

In recent years, several new diffusion MRI approaches have been proposed to explore microstructural properties of the white matter, such as Q-ball imaging and spherical deconvolution-based techniques to estimate the orientation distribution function. These methods can describe the estimated diffusion profile with a higher accuracy than the more conventional second-rank diffusion tensor imaging technique. Despite many important advances, there are still inconsistent findings between different models that investigate the “crossing fibers” issue. Due to the high information content and the complex nature of the data, it becomes virtually impossible to interpret and compare results in a consistent manner. In this work, we present novel fiber tractography visualization approaches that provide a more complete picture of the microstructural architecture of fiber pathways: multi-fiber hyperstreamlines and streamribbons. By visualizing, for instance, the estimated fiber orientation distribution along the reconstructed tract in a continuous way, information of the local fiber architecture is combined with the global anatomical information derived from tractography. Facilitating the interpretation of diffusion MRI data, this approach can be useful for comparing different diffusion reconstruction techniques and may improve our understanding of the intricate white matter network.

## Introduction

Diffusion MRI can be used to estimate the self-diffusion of water molecules in vivo (e.g., Moseley et al. [1]). Diffusion tensor imaging (DTI) describes the orientational preference of diffusion in the human brain, modeling it as a second-rank tensor [2]. Based on the diffusion tensor framework, virtual reconstructions of white matter (WM) fiber pathways can be created with fiber tractography (e.g., Basser et al. [3]). To convey more information than merely the fiber trajectories, alternative approaches to the conventional streamlines and -tubes have been proposed: hyperstreamlines, showing the second and third eigenvectors and eigenvalues of the diffusion tensor as the cross-sectional shape of the streamtube [4]; and streamribbons, where the local width of the ribbon indicates the coefficient of planar diffusion [5,6]. However, the additional information visualized in this way suffers from the same limitations inherent to DTI, i.e., the inability of the diffusion tensor to describe the diffusion correctly in regions of crossing or branching fiber tracts [7-12].

To overcome the limitations of DTI, several different methods have been proposed, e.g., Q-ball imaging (QBI) [13], constrained spherical deconvolution (CSD) [14], diffusion spectrum imaging (DSI) [15] – each having different requirements in terms of the acquisition protocol. QBI and DSI, on the one hand, have a direct mathematical relation between the measured diffusion signals and the estimated diffusion orientation distribution function (ODF) and diffusion propagator, respectively. The propagator obtained from DSI represents the 3D probability of spin displacement, where the diffusion ODF (dODF) calculated by QBI yields only the orientational aspect of the spin displacement [16]. On the other hand, CSD uses an estimated single fiber response function to deconvolve the DW intensities with this response function to obtain the fiber ODF (fODF), specifying the orientations of the underlying fiber populations. Tractography that makes use of these ODFs, which then is referred to as “multi-fiber” tractography, can take crossing fibers configurations into account, such as, for instance, in the centrum semiovale, where the cortico-spinal tracts (CST), arcuate fasciculus (AF), and lateral projections of the corpus callosum (defined in the remainder of this work as “latCC”) intersect one another [17-21].

With a growing number of diffusion MRI researchers opting for these more advanced diffusion MRI methods over conventional DTI, especially for tractography purposes, it is becoming increasingly important to fully utilize the potential offered by these approaches [22]. Despite the huge efforts invested in creating these new techniques, there are still several discrepancies and inconsistent findings between different algorithms (e.g., Jeurissen et al. [23]). To optimize the yield from diffusion data, it is important to understand the origins of these differences. In this context, data visualization plays an important role in facilitating the interpretation of high-dimensional diffusion MRI data. Visualization of the ODF is already possible on a local scale as voxel-wise glyph representations, which can be of great use to visualize the regional architectural complexity in known areas of crossing fibers [24]. However, as these local representations are only available for display at discrete positions [25-27], they are suboptimal to convey the anatomical continuity of fiber bundles. To date, and to the best of our knowledge, there are no fiber tractography visualization strategies that are specifically designed to address the interwoven and complex geometry of the WM fiber network [21,28,29].

In this work, we introduce an approach to visualize the local tissue architecture along reconstructed fiber tracts in a continuous way, creating hyperstreamlines for multi-fiber tractography data. This means that microstructural information from advanced diffusion methods is combined with large-scale anatomical information obtained from fiber tractography. With this new visualization method, the ODF is visualized along the fiber tracts to clearly illustrate the regions of crossing fibers. Additionally, by reducing the ODFs to their principal fiber orientations, streamribbons can be constructed to emphasize the orientations of the crossing fiber populations. The proposed visualization approaches create more complete representations of the microstructural architecture in fibers that pass through regions of complex WM configurations. As a result, these multi-fiber tractography visualizations may aid in our understanding of white matter organization. For clinical applications, improved visualization of local tissue microstructure along fiber tracts may benefit surgical planning (e.g., 30,31). For neuroscientific purposes, these visualizations could be used to compare results between multiple diffusion MRI methods, aiding in the understanding of their behavior in complex fiber architecture (e.g., 23).

## Materials and Methods

### Ethics statements

All subjects gave written informed consent to participate in this study under a protocol approved by the University Medical Center Utrecht ethics board.

### Tensor-Based Visualizations

**Figure 1:**
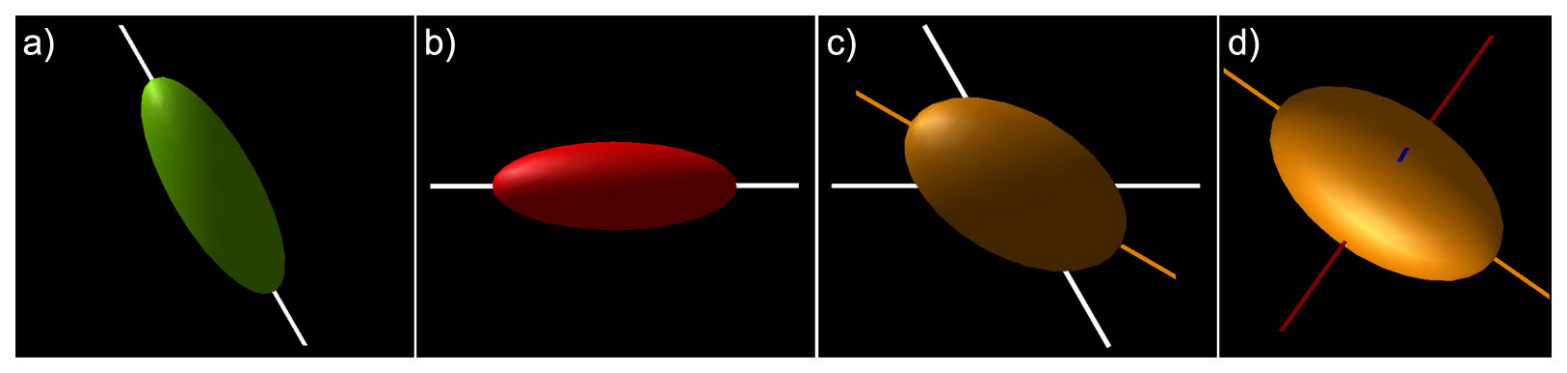
Conceptual difficulties of tensor-based visualizations of multi-fiber tractography data. In a) and b), two single fiber populations with their respective diffusion ellipsoids are shown. When these two coexist within one voxel (c), the first eigenvector (ε_1_ brown line) no longer corresponds to any of the underlying fiber orientations (white lines). d) The second (ε_2_ green) and third (ε_3_ blue) eigenvectors do not have a physical meaning with respect to the underlying fiber populations.

Before describing the multi-fiber visualization methods in detail, we will first discuss why current DTI based tract visualization approaches [4,6] are inappropriate for visualizing multi-fiber tractography data. Consider two fiber populations crossing at a 60° angle (Figure 1a,b). When these two populations coexist within one voxel – assuming slow exchange between these populations [7] – the resulting diffusion tensor can be estimated from the averaged diffusion weighted signals of the individual populations (as shown in Figure 1c). The first eigenvector of this tensor (**ε_1_**, brown line in Figure 1c) will be an average of the two fiber populations (white lines in Figure 1c). In DTI based tractography, the tract orientation will be determined by **ε_1_**, with **ε_2_** and **ε_3_** always perpendicular to **ε_1_** (Figure 1d). In the case of crossing fibers, **ε_1_** is not necessarily parallel to one of the fiber populations as determined by the multi-fiber diffusion method (Figure 1c). Consequently, **ε_2_** and **ε_3_** will not necessarily be perpendicular to the tract orientation for any of the two fiber populations, which means that the information visualized in this way will be misleading. The following will first detail the simulated fiber phantoms and in vivo data that will be used to demonstrate the concepts and benefits of our multi-fiber tractography visualizations. 

### Simulations

**Figure 2:**
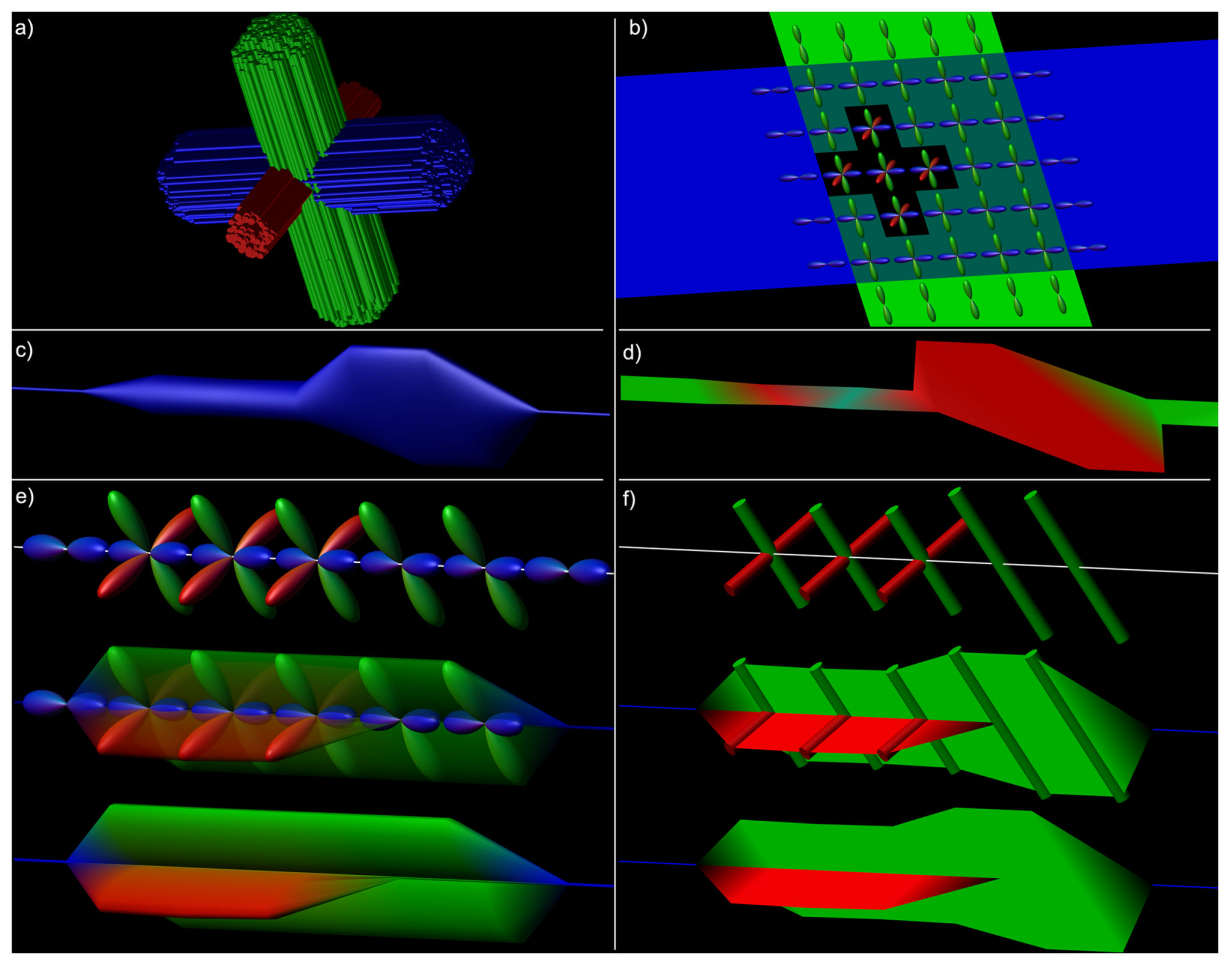
Hyperstreamlines and streamribbons in a simulated crossing fiber phantom. The global topology of a simulated fiber phantom is illustrated in a), with streamtubes color-encoded by the tract direction. The configuration was designed to contain distinct regions where two and three fiber populations intersect. fODF glyphs in this region clearly show these crossing populations (b). A single tract from the blue fiber bundle is shown in c) as tensor-based hyperstreamline and in d) as tensor-based streamribbon. In e), local fODF glyphs are shown along its trajectory, and a step-by-step creation of the hyperstreamline that envelops these glyphs. In f), the cylindrical glyph objects represent the distinct fODF peaks and the streamribbon is a continuous visualization of these peaks.

**Figure 3:**
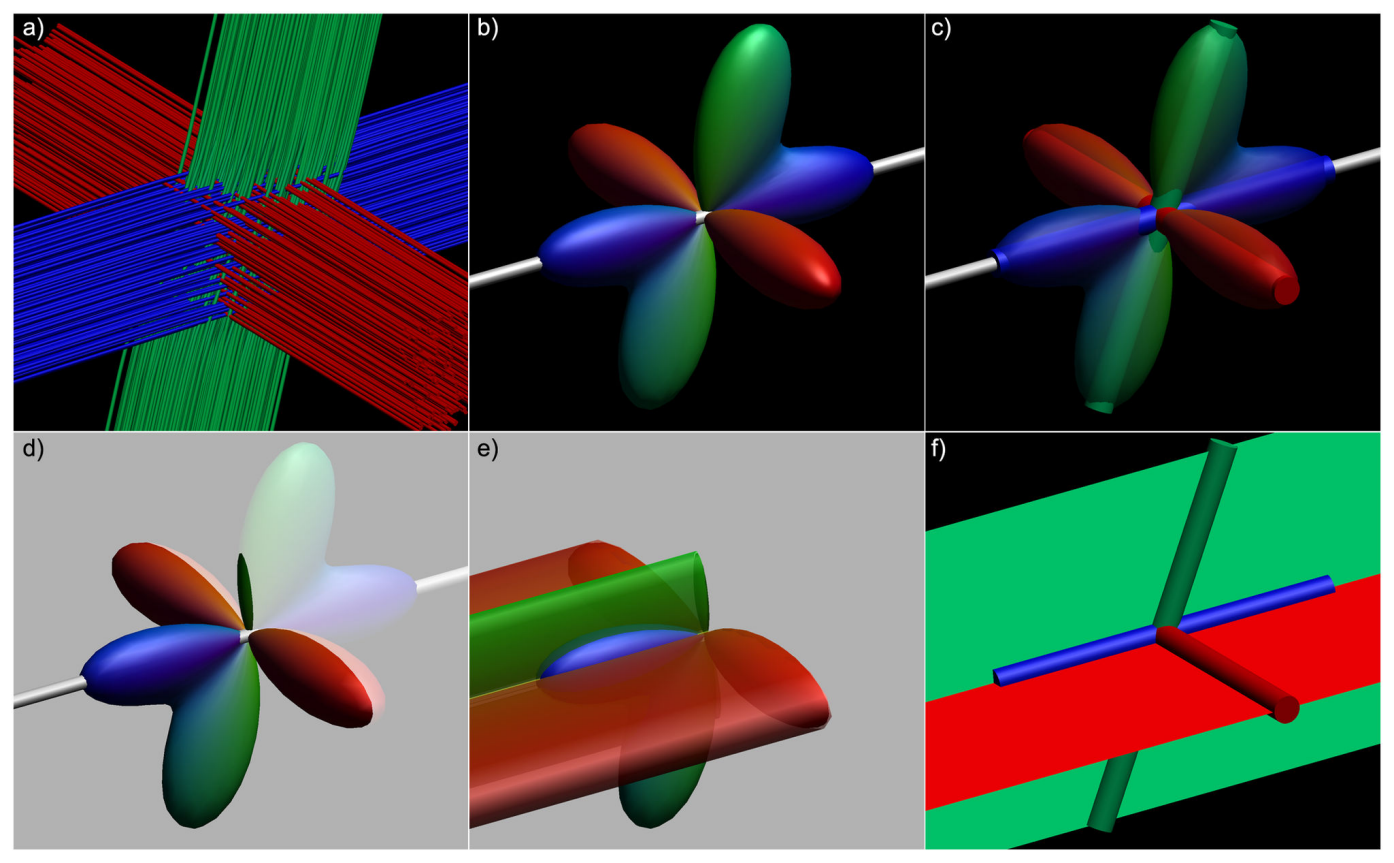
Difference between hyperstreamlines and streamribbons. A single tract-of-interest from a simulated fiber configuration (a) is created as hyperstreamline (b-e) and streamribbon (c,f). In b), the fODF at a location along this tract (white tube); c) fODF shown semi-transparently with its peak orientations; d) fODF shown with the plane perpendicular to the tract orientation, delineating the amplitude of the hyperstreamline; e) the hyperstreamline; f) streamribbons are created of the fODF peaks that do not correspond to the tract orientation.

To illustrate the concepts of the multi-fiber visualizations and to demonstrate the benefits over conventional tensor-based visualizations, we have created a toy example, where a configuration of three crossing neural fiber bundles has been simulated. It is designed in such a way that one bundle has a smaller cross-section, so that there are regions with both two and three fiber bundles intersecting each other, all crossing orthogonally (Figure 2a,b). To demonstrate the difference between the multi-fiber hyperstreamlines and streamribbons, another simulated phantom was designed in which not all populations cross at 90° angles (Figure 3a). Three fiber bundles were simulated, with two fiber populations crossing orthogonally (oriented inferior-superior and left-right) and one population oriented largely along the anterior-posterior axis. Defined in a spherical coordinate system, the orientations are: 1) inferior-superior, with azimuth angle θ=0° and polar angle φ=90°; 2) left-right, at θ=90° and φ=0°; and 3) mainly anterior-posterior, at θ=27° and φ=0°. Both phantoms were generated with the following parameters: 60 gradient directions, a b-value of 2500 s/mm^2^, and each single fiber population having a fractional anisotropy and mean diffusivity of 0.7 and 0.7×10^-3^ mm^2^/s, respectively [32]. For regions with multiple fiber populations, we assume that there is only slow exchange between these populations [7].

### In vivo diffusion MRI data

Diffusion MRI datasets were acquired from two healthy subjects (a 25 year old female and a 25 year old male) on a Philips 3T Achieva MR system (Philips Healthcare, Best, the Netherlands) using a single-shot spin echo EPI sequence, with one b=0 image and 60 diffusion-sensitizing directions acquired at a b-value of 2500 s/mm^2^ [33], using a SENSE acceleration factor of 2. The acquisition matrix of 112×112 was zero-filled to 128×128 with a field-of-view of 224×224 mm^2^, and 70 contiguous axial slices with thickness 2.0 mm were acquired. Sequence timings were TE = 107 ms, TR = 10.3 s and the average SNR for both datasets was 20 (estimated from regions in the centrum semiovale) [34].

Prior to data analysis, the datasets were corrected for eddy current induced geometric distortions and subject motion by realigning all diffusion-weighted images to the b=0 image using e*lastix* [35], with an affine transformation model and mutual information as the cost function [36]. In this procedure, the diffusion gradients were adjusted with the proper b-matrix rotation [37].

### Diffusion modeling and tractography

Examples of the proposed visualizations will be shown for two diffusion reconstruction methods: CSD [14] and QBI [13]. CSD estimates a response function of a single fiber population, assumes that all individual fiber populations are characterized by this function, and then deconvolves the acquired signal intensities to resolve the fODF (further details can be found in Tournier et al. [14]). In the QBI method employed, the acquired diffusion weighted signal is modeled using spherical harmonics, followed by the approximation of the dODF using the Funk-Radon transform [13]. Note that with these examples, we do not intend to provide a quantitative comparison between QBI and CSD, but rather demonstrate that our multi-fiber visualizations can be applied to different diffusion approaches. As such, standard QBI and CSD parameters were used as reported previously: Tuch [13] and Descoteaux et al. [38] for QBI; and Tournier et al. [14] and Jeurissen et al. [20] for CSD. Noteworthy is that, in contrast to the fODF (e.g., see 39,40), the dODF calculated with QBI is a spherical probability distribution, and thus normalized to unit probability over the sphere. To account for this difference, we have used two scaling approaches that yield comparable ODFs, both in their glyph representation and in their basis to form the hyperstreamline. For the dODF, standard “min-max” scaling was used, as proposed by Tuch [13]. For the fODF scaling, we have used a similar scaling, here called “normalization scaling”, where the amplitudes of the fODF in each voxel are scaled such that the maximum amplitude is equal to one. Deterministic fiber tractography was performed using the peak fiber orientations of the ODFs, where the number of unique fiber populations at each location, and their orientations, were computed as described in Jeurissen et al. [23]. ODF peak thresholds were defined as 10% of their maximum value. The maximum number of unique fiber populations was limited to three for all analyses. Fiber tractography using both methods was performed with the *ExploreDTI* diffusion MRI toolbox [41] (www.exploreDTI.com).

### Multi-fiber tractography visualization

The conventional representations of fiber pathways are either streamlines (e.g., Basser et al. [3]) or streamtubes [4,42]. Hyperstreamline and streamribbon approaches were originally designed with the purpose of visualizing the second and third eigenvectors and eigenvalues of the diffusion tensor [4,6]. With the tensor unable to characterize crossing fibers, the tensor-based hyperstreamlines cannot correctly visualize the local architecture. In the following sections, we will present the new multi-fiber tractography visualizations.

#### Multi-fiber hyperstreamlines

For DTI-based hyperstreamlines, the cross-sectional shape of the hyperstreamline at each tract position is determined by the orientation of the second and third eigenvectors (scaled as a function of the eigenvalues) of the diffusion tensor [4]. In the following, we extend this concept for diffusion MRI data with a higher angular resolution, where the cross-sectional shape is defined by the ODF, instead of the tensor. For the “multi-fiber hyperstreamline”, the local shape of the hyperstreamlines is then determined by the three-dimensional ODF profile. At each tract position, **r**, we define a circle with its center in **r** and lying in the plane perpendicular to the tract orientation, **t**. The vectors from **r** to the points on this circle are then scaled by the ODF amplitude along these orientations to delineate the cross-section of the hyperstreamline . For a more detailed description, please refer to the pseudo-code in Appendix S1. An example hyperstreamline of the simulated fiber phantom of Figure 3a is shown in Figure 3b-e. Notice that, by visualizing the ODF amplitude perpendicular to the tract orientation, the maximum ODF amplitude crossing the tract is not displayed if populations cross at other angles than 90°.

Appendix S3 describes the computational costs of these hyperstreamlines in more detail.

#### Multi-fiber streamribbons

Tract ribbons have initially been proposed to visualize the axial asymmetry of the diffusion tensor [6]. In this work, we introduce “multi-fiber streamribbons” by reducing the ODFs along the tract to their principal peak orientations. In this way, streamribbons can be used for efficient visualization with a lower rendering complexity than streamtubes or hyperstreamlines [6,24]. For each ODF peak orientation that does not correspond with the tract orientation, a streamribbon is created – as shown in Figures 2f and 3c,f – which means that in the case of three unique peaks, two streamribbons are created. For each existing ribbon, the angular deviation is calculated from the current ribbon orientation to the ODF peak or peaks at the next vertex along the tract. The ribbon is then connected to the peak that deviates the least. In those voxels where the ODF indicates a single fiber population, the streamribbons converge naturally to a streamline (Figure 2f). Appendix S2 presents pseudo-code on how the streamribbons are created.

Appendix S3 describes the computational costs of these hyperstreamlines in more detail.

#### Color-encoding

The conventional coloring of streamlines and -tubes shows the tract orientation at each vertex, providing additional visual cues about the tract orientation such as the “direction encoded color” (DEC) map proposed by Pajevic and Pierpaoli [43]. This coloring according to the diffusion orientation can still be used for our proposed visualizations, further enhancing the interpretation of the local microstructural organization. To this end, we propose such color-encoding scheme, termed ‘ODF color-encoding’, where:

• All tract vertices with a single fiber population are colored according to the tract direction;
• Vertices with two unique fiber populations, where one population corresponds to the tract direction, are colored according to the orientation of the population crossing the tract;
• Vertices with three or more populations have the individual points on the hyperstreamline circumference colored by the orientation of that point with respect to the tract vertex.

As an alternative to the proposed ODF color-encoding, where the orientations of the fiber populations are highlighted, it is also possible to highlight the number of fiber populations. This can be used to distinguish between regions with one, two, or more than two fiber populations more clearly (as also shown in Jeurissen et al. [23]).

### In vivo tract visualizations

In vivo examples of multi-fiber tractography visualization are shown for fiber tracts from the lateral projections of the corpus callosum (abbreviated as latCC), the corticospinal tract (CST), and the arcuate fasciculus (AF), all of which are known to intersect other WM structures [44]. In addition, tracts from the bilateral cingulum bundles are shown, as an example of fiber bundles with a relatively small cross-sectional area. To maintain the focus on the additional information conveyed by our new visualization approaches, these examples are only shown for the CSD results. For a specific configuration of AF pathways, however, we provide an example illustrating that both QBI and CSD based tractography results can benefit from multi fiber visualizations.

## Results

### Simulations

Figure 2a shows an example fiber configuration, where there are regions with single fiber populations as well as regions with two and three fiber populations crossing, as can also be seen from the fODF glyphs (calculated from CSD) shown per voxel in Figure 2b. The tensor-based hyperstreamline and streamribbon are shown in Figures 2c and 2d, respectively. Creation of the hyperstreamline is shown in Figure 2e. When displayed at each vertex of the fiber tract, the fODF glyphs in Figure 2e illustrate the local microstructure, with one or two populations crossing the fiber tract, depending on the location along the tract – as can be verified from Figures 2a and 2b. The step-by-step creation of the corresponding hyperstreamline is also shown in Figure 2e, where the ODF color-encoding further highlights the architectural organization. The multi-fiber streamribbon is created by continuously connecting the peak orientations and magnitudes of the populations crossing the fiber tract. In Figure 2f, the peak orientations are shown along the tract as cylinders with the streamribbons connecting these peaks and for two different color-encodings proposed in this work. Notice that the tensor-based visualizations (Figures 2c and 2d) show some contrast along the tract between regions with one, two, and three fiber populations, but they do not visualize the orientations of the underlying fiber populations in an unambiguous way. The multi-fiber based visualizations, on the other hand, do show the fiber orientations very clearly, both as hyperstreamline and as streamribbons.

The simulated fiber phantom in Figure 2 has three fiber populations that are oriented perpendicularly. Thus, the peak orientations were perpendicular to the tract direction, where the hyperstreamline envelops the full ODF profile (Figure 2e). However, if populations are no longer orthogonal, the ODF profile perpendicular to the tract no longer describes the peak amplitude of the ODF. As a result, the hyperstreamline and streamribbons show different complementary characteristics of the fiber populations (as can be seen when comparing Figure 3e and 3f).

### In Vivo Tract Visualizations

In the following subsections, in vivo examples of fiber bundles that pass through regions of complex fiber architecture are shown, demonstrating the additional information visualized with these new multi-fiber tractography representations.

#### Corpus callosum

**Figure 4:**
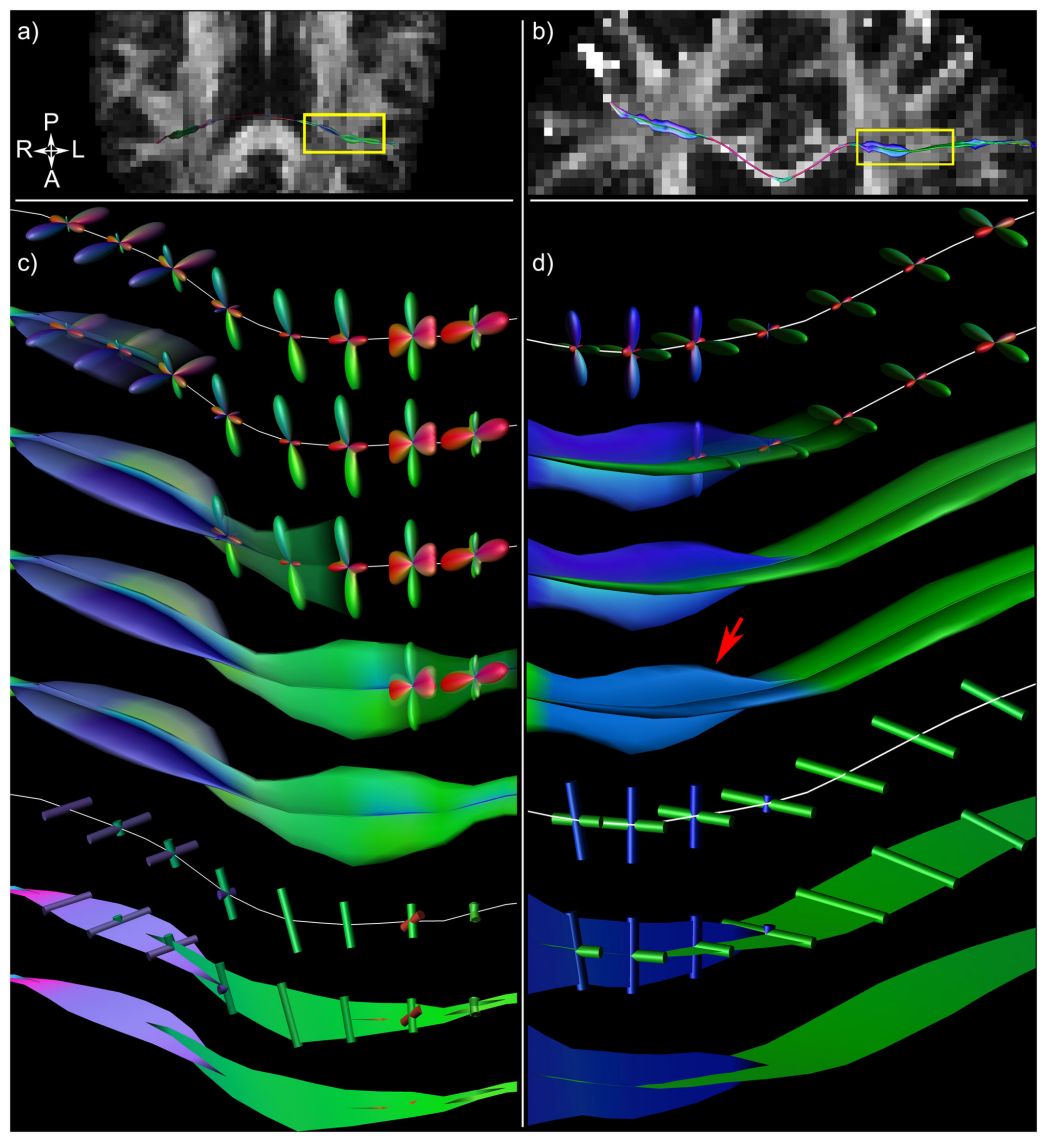
Hyperstreamlines and streamribbons for a tract from the corpus callosum. Axial (a) and coronal (b) slices of the brain of the two subjects (subject one: a) and c); subject two: b) and d)) are shown, with a tract from the lateral projections of the corpus callosum (latCC) visualized as multi-fiber hyperstreamline. The boxed regions in a) and b) are enlarged in c) and d), respectively, showing the construction procedures of the hyperstreamline and streamribbon. Starting from the middle of the brain, the fiber tracts first cross the cortico-spinal tracts (CST) before intersecting the arcuate fasciculus (AF) more laterally. From the visualizations in c) and d), regions with three distinct fiber populations can be seen along the tracts, i.e., where the AF and CST both cross the latCC pathway. This is highlighted when color-encoding the hyperstreamline by the number of ODF peaks (red arrow).

Fiber tracts from the lateral projections of the corpus callosum (latCC) are displayed in Figure 4. Hyperstreamlines are shown for subject one in an axial orientation (a) and for subject two in a coronal orientation (b); with the tract segment in the boxed regions enlarged in c) and d), respectively. Figure 4c,d also show that the ODF amplitudes perpendicular to the fiber tract pathway provide the cross-sectional shape of the multi-fiber hyperstreamline. Video S1 shows this creation process in more detail. When starting at the mid-sagittal region, the tract pathway first crosses the CST and then the AF, both clearly depicted in the ODF glyphs and the hyperstreamlines as the (blue/purple) inferior-superior and (green) anterior-posterior fiber populations, respectively. As can most clearly be observed in the hyperstreamline that is colored by the number of ODF peaks – indicated by the red arrow in d) – there are regions along this tract with three fiber populations. In c) and d), these tract segments can also be observed as the multi-fiber streamribbons. 

#### Cortico-spinal tracts

**Figure 5:**
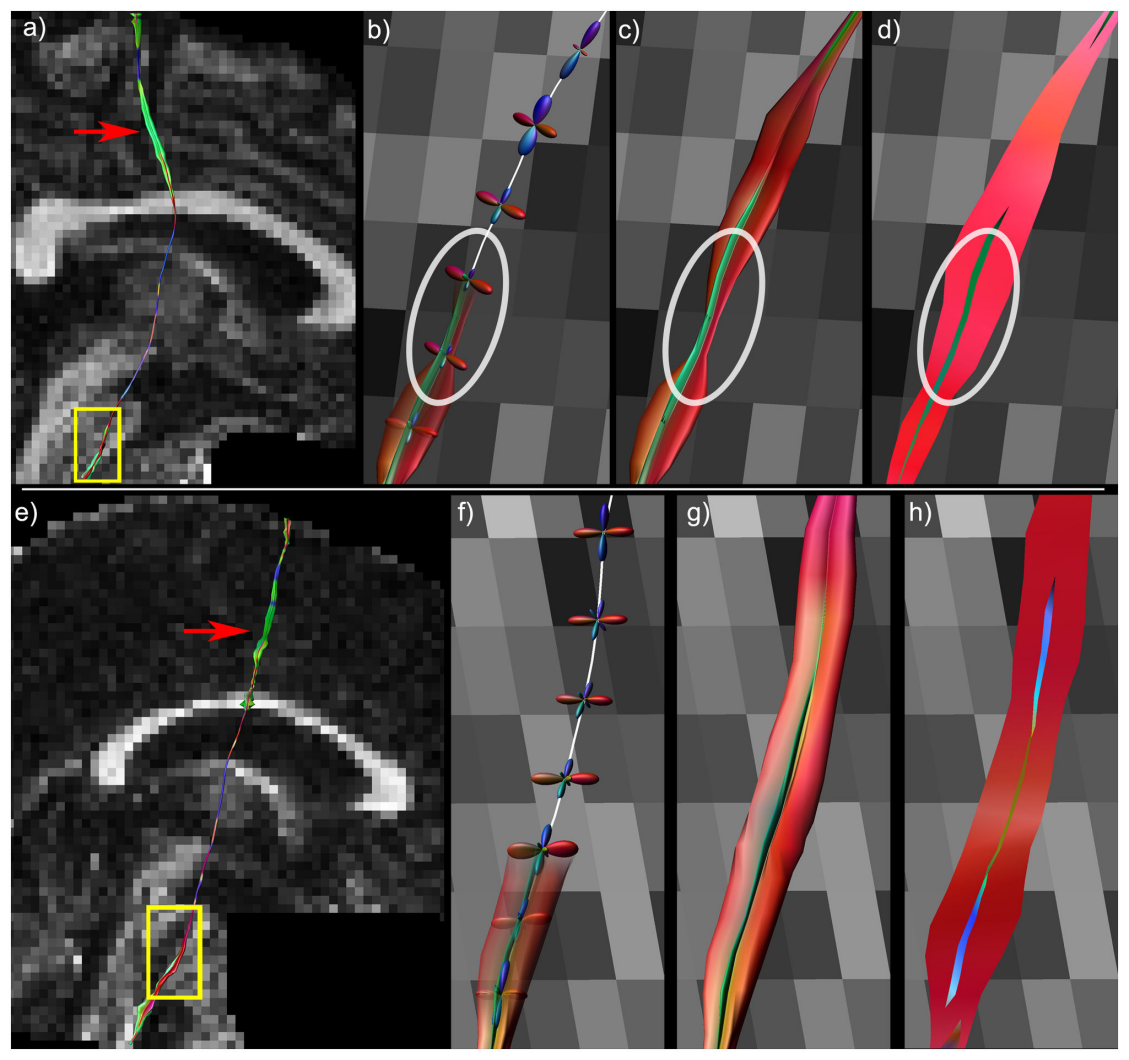
Hyperstreamlines and streamribbons for a tract from the cortico-spinal tract. A global overview of the mid-sagittal slice of the brain with hyperstreamline is shown in a) for one subject. A segment of the cortico-spinal tract (CST) in the region of the pons – indicated on a) by the yellow rectangle – is enlarged in b-d). The multi-fiber hyperstreamline is shown in b) and c), indicating left-right (LR, red) and anterior-posterior (AP, green) oriented fiber populations. This can also be seen from the streamribbon representation in d). The white ellipses (b-d) highlight a region where the LR-oriented fibers are not perpendicular to the tract pathway, resulting in a hyperstreamline that does not reflect the full ODF amplitude, whereas the streamribbons do. For the second subject, f-h) show the multi-fiber hyperstreamline and streamribbon representations of a small segment of the tract pathway indicated by the yellow rectangle on e). Note that more superior along the CST one can detect prominent AP-oriented fiber populations that correspond to the arcuate fasciculus (red arrows in a and e).

Fiber tracts from the CST are shown in Figure 5. Global overviews of the mid-sagittal slice of the brain with the hyperstreamline are shown in a) for subject one and e) for subject two. Figure 5b,c shows how a small segment of the tract in the region of the pons – see yellow rectangle in a) – is visualized as a multi-fiber hyperstreamline. In d), the corresponding streamribbon is illustrated. For subject two, the hyperstreamline and streamribbon are shown in e-h). According to the known anatomy in this region, there are clear indications for left-right oriented transverse pontine fiber populations (also seen in Tournier et al. [10] and Aggarwal et al. [45]) and front-back oriented fiber populations of the middle cerebellar peduncle [46]. More superiorly along the CST one can also clearly observe anterior-posterior oriented fiber populations crossing the tract, indicated by the red arrows in a) and e).

#### Arcuate fasciculus

**Figure 6:**
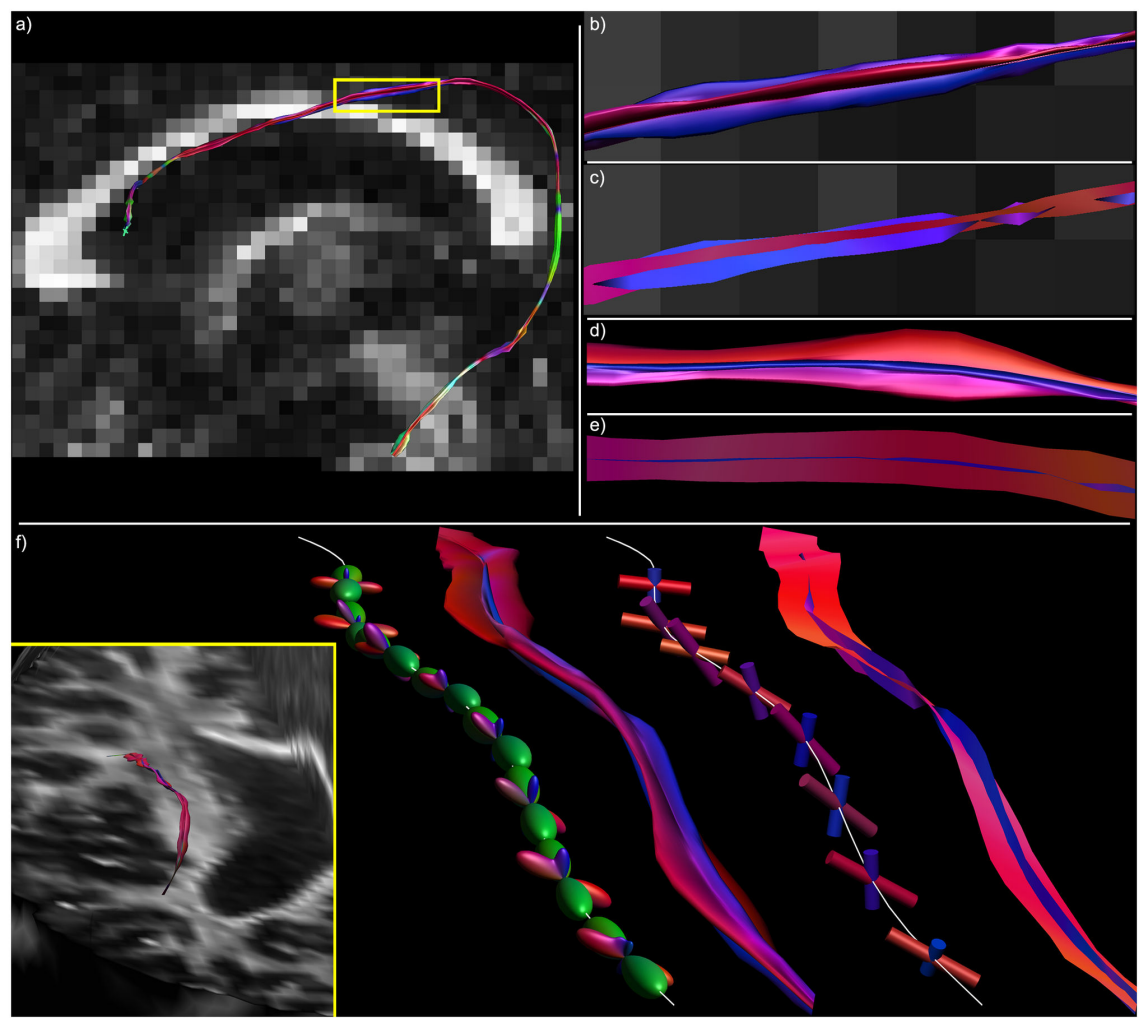
Hyperstreamlines and streamribbons for a tract from the arcuate fasciculus. a) An example of the multi-fiber hyperstreamline and streamribbon is shown for a single tract of the arcuate fasciculus (AF). Tract segment indicated by the yellow rectangle in a) is shown in a sagittal (b,c) and axial (d,e) view, for both the hyperstreamline (b,d) and streamribbon (c,e). The same segment is shown in f) from a near coronal view angle, indicating (from left to right): the ODF glyphs along the tract; the corresponding multi-fiber hyperstreamline; the glyph objects representing the ODF peak orientations and magnitudes; and the corresponding multi-fiber streamribbon. The inset depicts the oblique viewing angle. Right-left and inferior-superior oriented fiber populations are visible crossing the AF and represent the lateral projections of the corpus callosum and the cortico-spinal tracts, respectively.

Figure 6 shows a multi-fiber visualization of the left arcuate fasciculus (AF). The hyperstreamline and streamribbon representations both illustrate the orientations of the fiber populations crossing the AF in accordance with the known local architecture. Enlargements of the indicated region (yellow rectangle in a)) are shown sagittally (b,c) and axially (d,e), clearly indicating that there are two other fiber populations (latCC in reddish and CST in bluish) crossing the AF in this region. An oblique view of the same tract segment is presented in f), showing the amplitudes of the fiber populations of the latCC and CST and their continuity along the tract pathway in more detail. There are also anterior-posterior oriented fiber populations intersecting the AF at its more posterior part, corresponding with the inferior fronto-occipital fasciculus [46] (Figure S1). Video S2 shows this hyperstreamline in full detail. 

**Figure 7:**
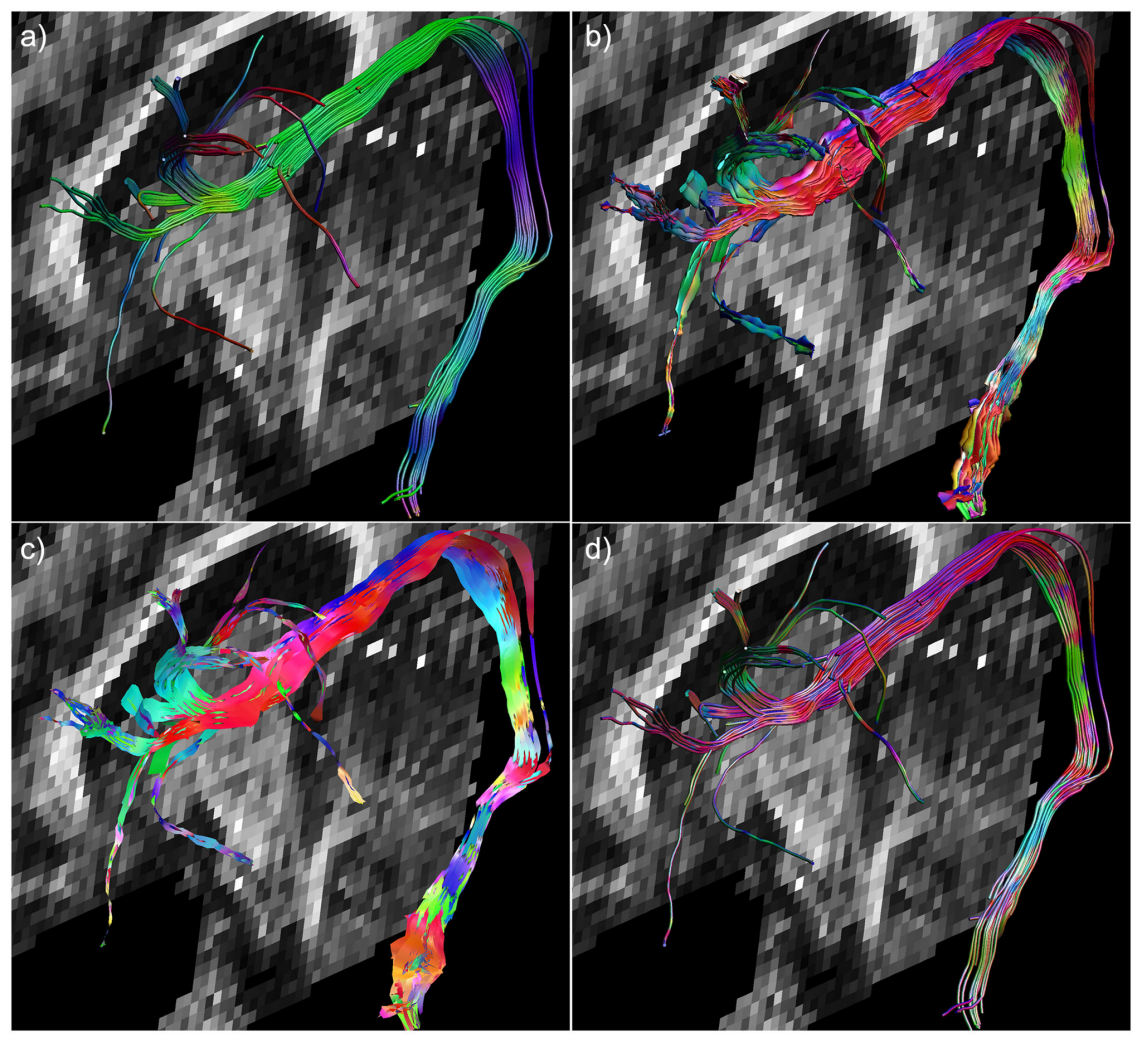
Arcuate fasciculus visualized as streamtubes with different color-encoding. In a), the arcuate fasciculus (AF) is shown with its conventional streamtube representation and with color-encoding according to the tract direction. The multi-fiber hyperstreamline and streamribbon with the orientation color-encoding according to the ODF are shown in b) and c), respectively. Standard streamtubes have been visualized in d), but then using the ODF-based color-encoding used in b).

To demonstrate that multi-fiber tractography visualizations can depict the microstructural architecture not only for a single tract pathway but also for whole fiber bundles, Figure 7 shows a comparison of the left AF between standard streamtubes (a), multi-fiber hyperstreamlines (b), and streamribbons (c) with orientation color-encoding. In d), the conventional streamtube representation is used, but with the orientation color-encoding from the ODF as shown in b). 

**Figure 8:**
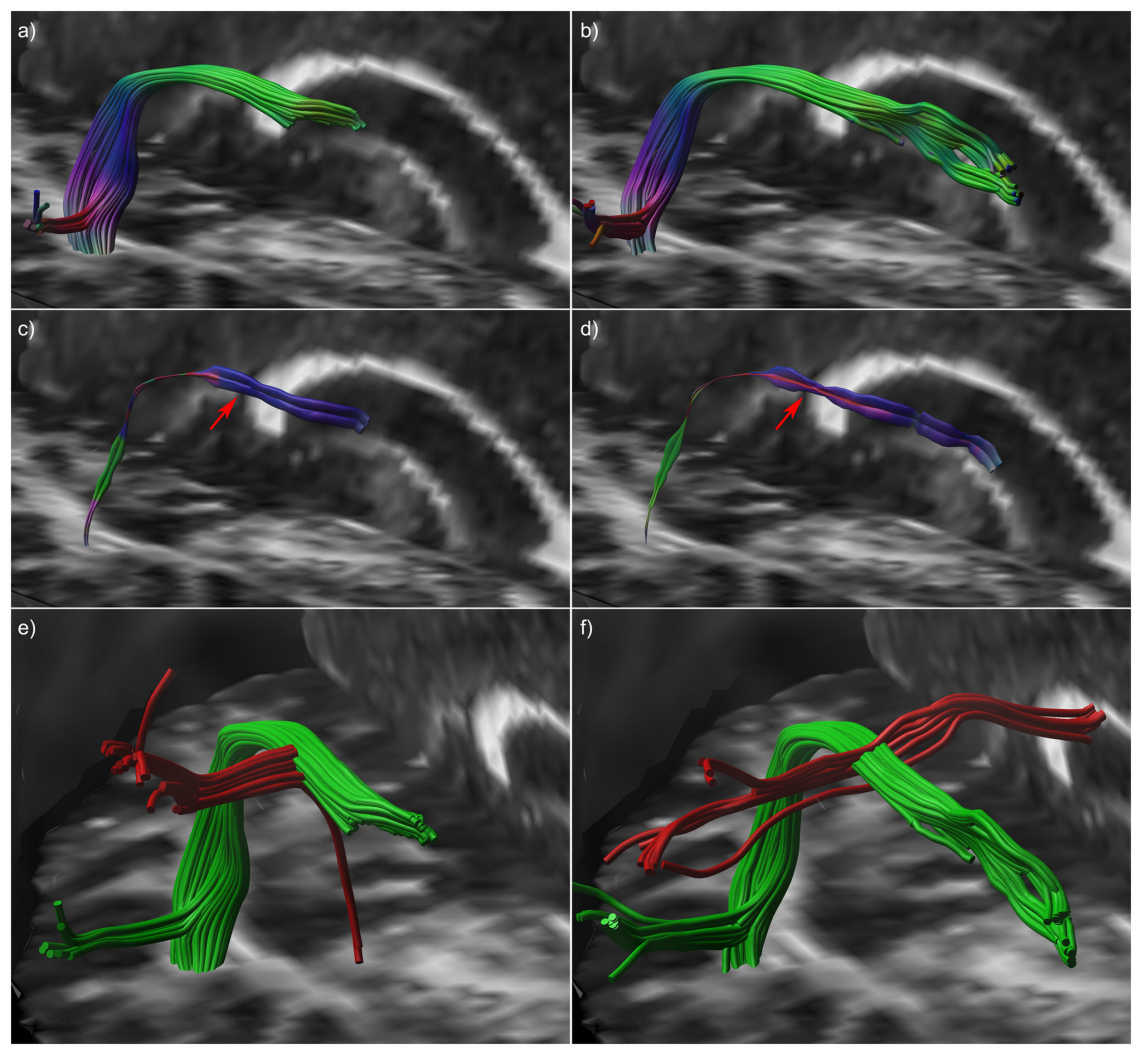
Example comparison of hyperstreamlines created using two HARDI methods. Hyperstreamline examples to compare different methods. The reconstructed arcuate fasciculus (AF) is shown for QBI (a) and for CSD (b). Single tracts from the QBI bundle and CSD bundle are shown as a multi-fiber hyperstreamline in c) and d), respectively. This visualization, both in cross-sectional shape as well as color-encoding, highlights an important difference between the two used methods. For CSD (d), clear left-right fiber populations (red) can be seen crossing the AF (indicated by the arrow), whereas QBI (c) does not display these populations: these lateral projections of the corpus callosum (latCC) could not be detected with QBI. Verification can be seen in e) and f), where the AF is shown in green and the latCC in red (seeded lateral to the AF it should target the corpus callosum, as seen in f).

When generating tractography data with different methods, subtle differences between them can be observed using the proposed visualization approaches (as illustrated in Figure 8). A part of the right AF is shown with QBI-based tractography (a) and CSD-based tractography (b) with the conventional tract representation. For each approach, a single pathway is shown as a QBI hyperstreamline (c) and a CSD hyperstreamline (d), highlighting the subtle differences in ODF reconstruction between QBI and CSD in the superior portion of the AF as indicated with the red arrow. In e) and f), the AF pathways (green) are shown in combination with the post-hoc reconstructed latCC trajectories (red) in the region of the red arrow, confirming the intersection of (part of) the latCC pathways.

#### Cingulum

**Figure 9:**
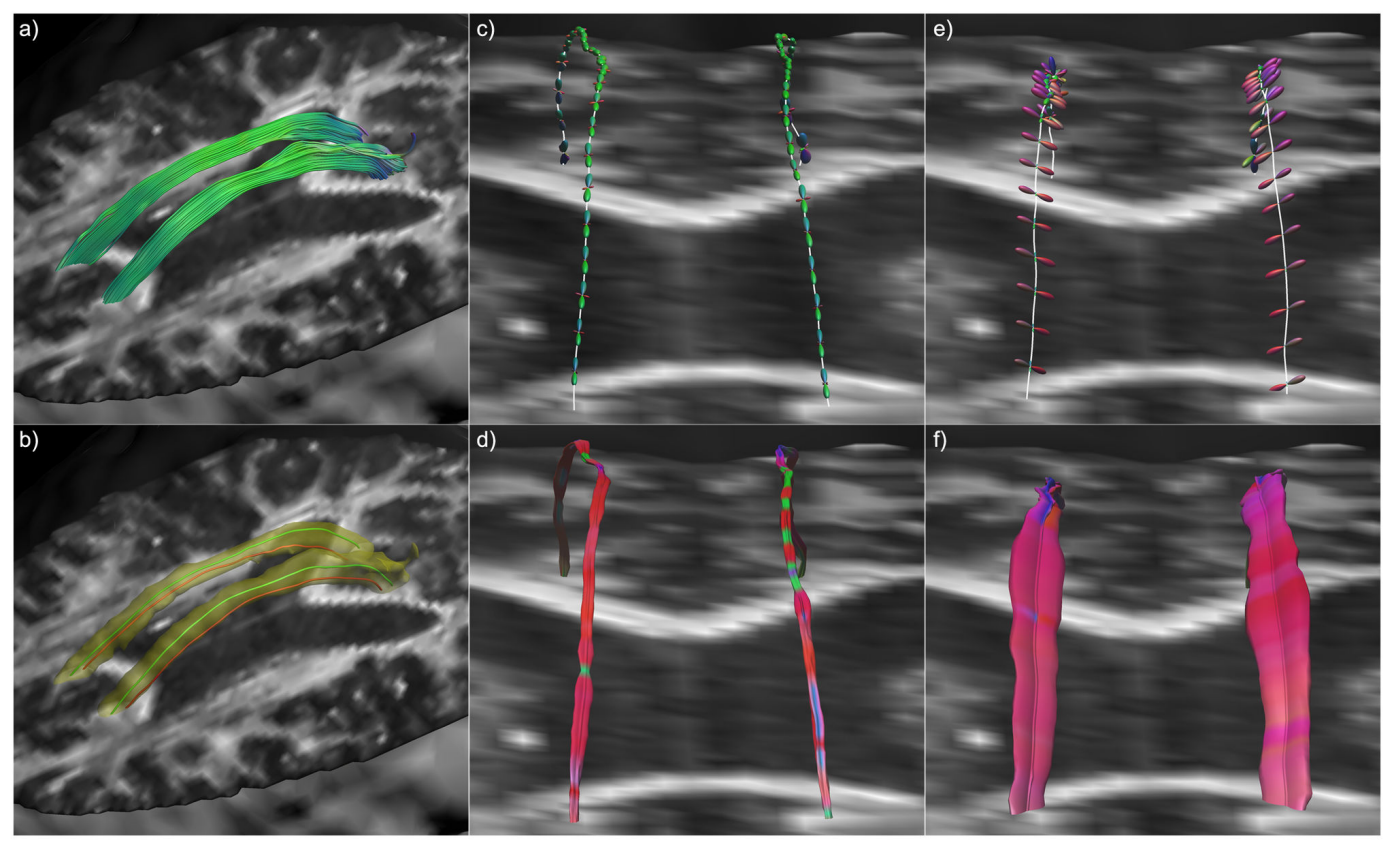
Hyperstreamlines and streamribbons for a tract from the cingulum. Superior segment of the bilateral cingulum bundles (a). From these full bundles – shown in b) as a yellow haze for anatomical reference – one tract has been selected at the center of each bundle (shown in green in b) and one tract at the interface of the corpus callosum (red in b). These “center” (c and d) and “edge” (e and f) tracts have been with the fODF glyphs along their trajectories (c and e) and as hyperstreamlines (d & f). Strong difference between the magnitudes of the left-right populations can be observed between (c) - (d) and (e) - (f). From the ODFs in c) it is difficult to interpret the continuity of orientation and magnitude of the left-right populations; whereas this is very easy from the hyperstreamlines in (d).

For the bilateral cingulum bundles (Figure 9a), one tract pathway has been selected towards the bottom of the cingulum, i.e., an “edge” tract located more towards the interface with the corpus callosum (red in Figure 9b), and one tract pathway that is located more at the “center” of the bundles (green in Figure 9b). These “center” and “edge” tracts are shown in Figures 9c,d and 9e,f respectively, from a frontal-superior angle with the same hyperstreamline settings. A strong difference in the width of the hyperstreamlines can be observed between the center (d) and edge (f) tracts, reflecting a marked difference in fiber architecture in these different regions of the cingulum. Notice that even the “center” tract shows left-right oriented populations along most of its trajectory. 

## Discussion

In this work, we have developed multi-fiber tractography visualization strategies that combine information of local white matter fiber architecture – e.g., the information captured by the dODF or fODF – with global anatomical information from fiber tractography, so as to create more complete representations of the microstructural organization of the WM. These visualizations will be made freely available to the community, as part of the *ExploreDTI* diffusion MRI toolbox [41] (www.exploredti.com).

The two proposed approaches, multi-fiber hyperstreamlines and multi-fiber streamribbons, visualize different properties of the ODFs, with the most pronounced difference in situations where fiber populations are not orthogonal (as illustrated in Figure 3). In vivo, when fiber population orientations will generally not be orthogonal, the two methods can be used to visualize complementary information from the ODF. An example of this can be observed in the pons, highlighted by the white ellipse in Figure 5b-d. From the streamribbon (d), it is clear that the magnitudes of the fiber populations in the shown segment do not differ much. This can be verified from the ODF glyphs visualized along the tract (b), but this is not apparent in the hyperstreamline (c). In the superior part of the AF (Figure 6d,e), a similar difference in visualization of ODF properties can be seen between the hyperstreamline and the streamribbon. In general, for interpretation of the ODF peak magnitudes, the streamribbons are preferable. However, due to the two-dimensional nature of a ribbon, investigating the specific configuration of the streamribbons can be non-trivial. By reducing of the full ODF to their peak orientations, the information about the shape of the peaks is discarded, making it impossible to distinguish between sharp or very broad ODF peaks. The three-dimensional nature of the hyperstreamlines lends itself to more intuitive interpretations of the local architecture. In some situations, for instance in the absence of free water diffusion, the fODF is modeled as a very spiky function (e.g., 47,48). In such cases, where fODF lobes resemble delta-peaks, the hyperstreamlines essentially “collapse” to streamribbons.

By comparing multi-fiber hyperstreamlines of fiber tracts reconstructed with two different approaches to estimate the ODF, we have demonstrated that this method can be used to show subtle differences between the results of the two methods that with conventional fiber tractography visualizations would have gone unnoticed. In Figure 8, for instance, the latCC pathways were interdigitating the AF in only one of the diffusion methods as suggested by the hyperstreamlines. The validity of this observation was backed up by finding a difference in configuration of the latCC pathways between both approaches. However, it should be clear that in this work, it was not our aim to contrast different diffusion reconstruction methods in terms of “accuracy” or “validity”, but purely to illustrate that the proposed multi-fiber hyperstreamlines can be an exploratory means to investigate these methods more intuitively.

The proposed multi-fiber hyperstreamlines and streamribbons can be extended to any multi-fiber diffusion model. For instance, one could opt to display the ball-and-multiple-stick model [17] using a streamribbon for each of the “stick” compartments when more than one “sticks” are present. Alternatively, the multiple-tensor approach [49] can be visualized by combining those diffusion ellipsoids that do not belong to the tract itself as hyperstreamlines or by using the principal diffusion directions to construct streamribbons.

When one is presented with a global view of an entire fiber bundle as in Figure 7, distinguishing the shape of the individual hyperstreamlines becomes complicated. The amount of information in these hyperstreamlines and streamribbons could result in an image that is too cluttered for easy interpretation. As a more subtle alternative to visualize local architectural information, without making the images too complex to interpret, the hyperstreamlines can be reduced to simple streamtubes, while maintaining the ODF color-encoding. In addition, streamtubes have a lower computational complexity compared with the multi-fiber hyperstreamlines, which could be another motivation to use the conventional streamtube shape. Video S3 shows the CST visualized as streamtubes (with the conventional color-encoding (left) and ODF color-encoding (right). The ODF color-encoded streamtubes show the presence, location, and orientation of crossing fiber populations along the CST, displaying only a single fiber population in the region of the internal capsule.

The initial hyperstreamline and streamribbon approaches were designed with the purpose of visualizing the diffusion tensor in more detail [4,6]. Hyperstreamlines have also been used to convey information on the uncertainty in fiber orientation, where the width of the hyperstreamline represents the local “cone of uncertainty” of the first eigenvector [9,50]. Alternatively, streamsurfaces have been proposed to visualize regions of planar diffusion as surface structures [4]. For instance, at interfaces between fiber bundles where partial volume effects cause the diffusion profile to be more planar in shape, these streamsurfaces create a virtual delineation of the edges of these fiber bundles, aiding in the distinction between different fiber structures. However, measures of planar diffusion, as used in these streamsurfaces and in conventional streamribbons, are not specific for the underlying tissue microstructure and are heavily affected by the presence of crossing fiber architecture [12]. By contrast, the proposed multi-fiber hyperstreamlines and streamribbons give more specific representations of the microstructural information obtained from diffusion MRI data.

**Figure 10:**
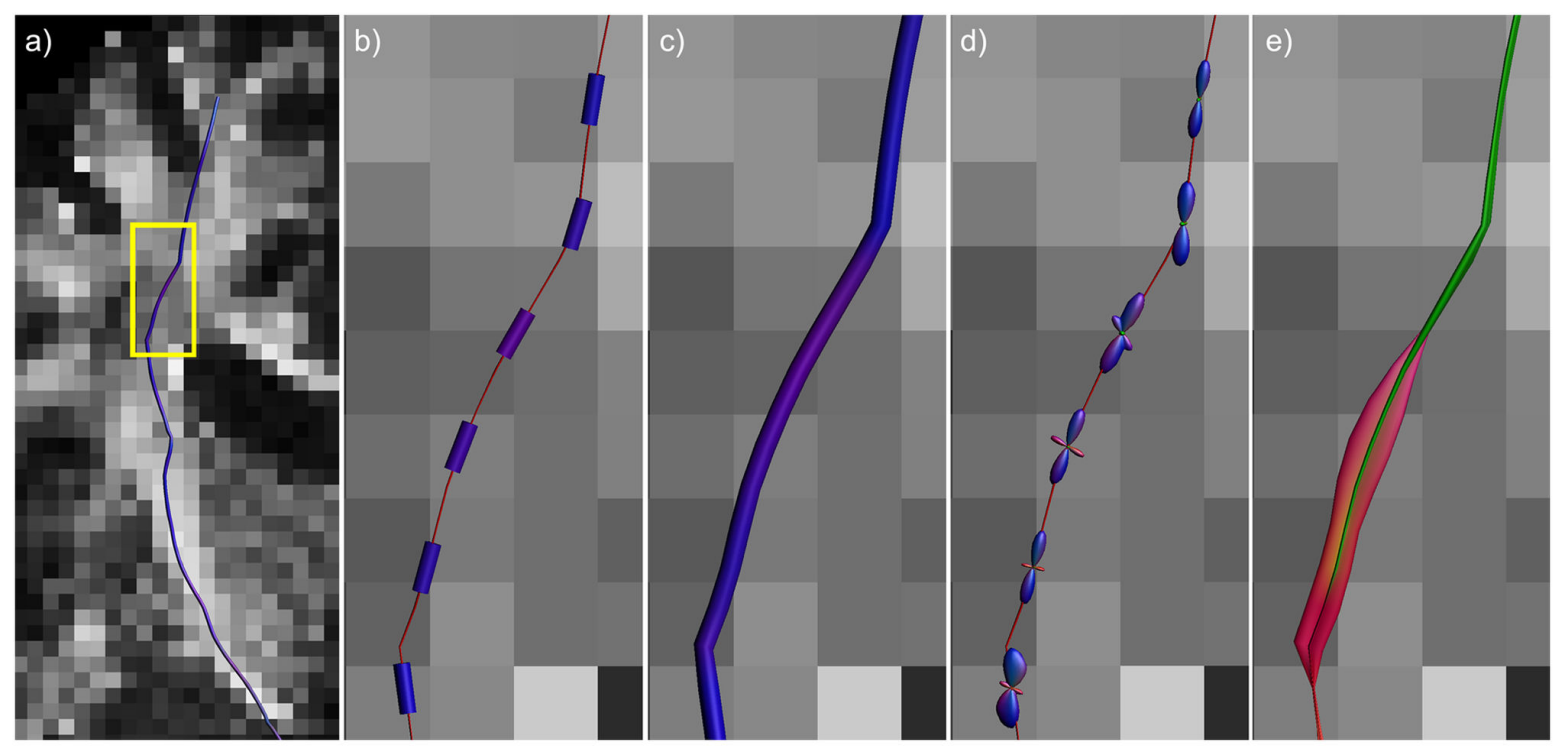
The difference between discrete and continuous visualization. A coronal overview of the tract to be visualized is seen in a), with the main eigenvector at discrete locations along the tract (b) and the tract itself (c). Analogous to b) and c), d) and e) show the fODF glyphs at discrete locations along the tract and the multi-fiber hyperstreamline, respectively. The continuity is difficult to grasp from the glyphs alone (b and d), whereas the continuous representations (c and e) give a more intuitive feel, are easier to interpret, and represent a more complete picture of the underlying WM pathway.

Previous work on visualization of multi-fiber diffusion data has mainly focused on efficiently displaying the ODF glyphs not only locally but in larger regions of the brain. Interpretation of ODF profiles of each voxel in an entire imaging slice can become difficult by the immense amount of data visualized. Reducing the ODF to its peak orientations yields more easily interpretable data in such situations, with the additional benefit of being less memory-consuming and more rendering-efficient [24]. With the development of advanced interactive and exploratory tools for visualizing ODF glyphs, however, it has become possible to visualize the full ODFs efficiently in large quantities, e.g., for an entire slice [26,51]. Recent work has focused on representing the ODF glyphs at discrete points along a tract [25,27], whereas the multi-fiber hyperstreamlines proposed in this work provide a continuous and, hence, a more intuitive representation of the data. To further emphasize this difference, an example of the discrete and continuous way to visualize a fiber trajectory is given in Figure 10 using both DTI-based and multi-fiber reconstructions. Plotting the first eigenvectors yields the discrete version of a reconstructed tract, but the tract itself gives a continuous and, therefore, more intuitive representation of the data. This is also the case for the multi-fiber hyperstreamline, which – being also continuous – gives a more complete view compared to showing ODF glyphs only at discrete locations along the tract pathway. This observation is supported by the in vivo examples shown in Figures 4c,d, 6f, and 9, where data interpretation for one continuous 3D object, the hyperstreamline, is more straightforward than for the individual 3D objects, which can obscure each other. Furthermore, as can be seen in Figures 4a,b, 5a,e, hyperstreamlines can directly pinpoint the main areas of crossing fibers, where individual glyphs are less powerful. This observation can also be made in Figure 2b of Kezele et al. [25].

**Figure 11:**
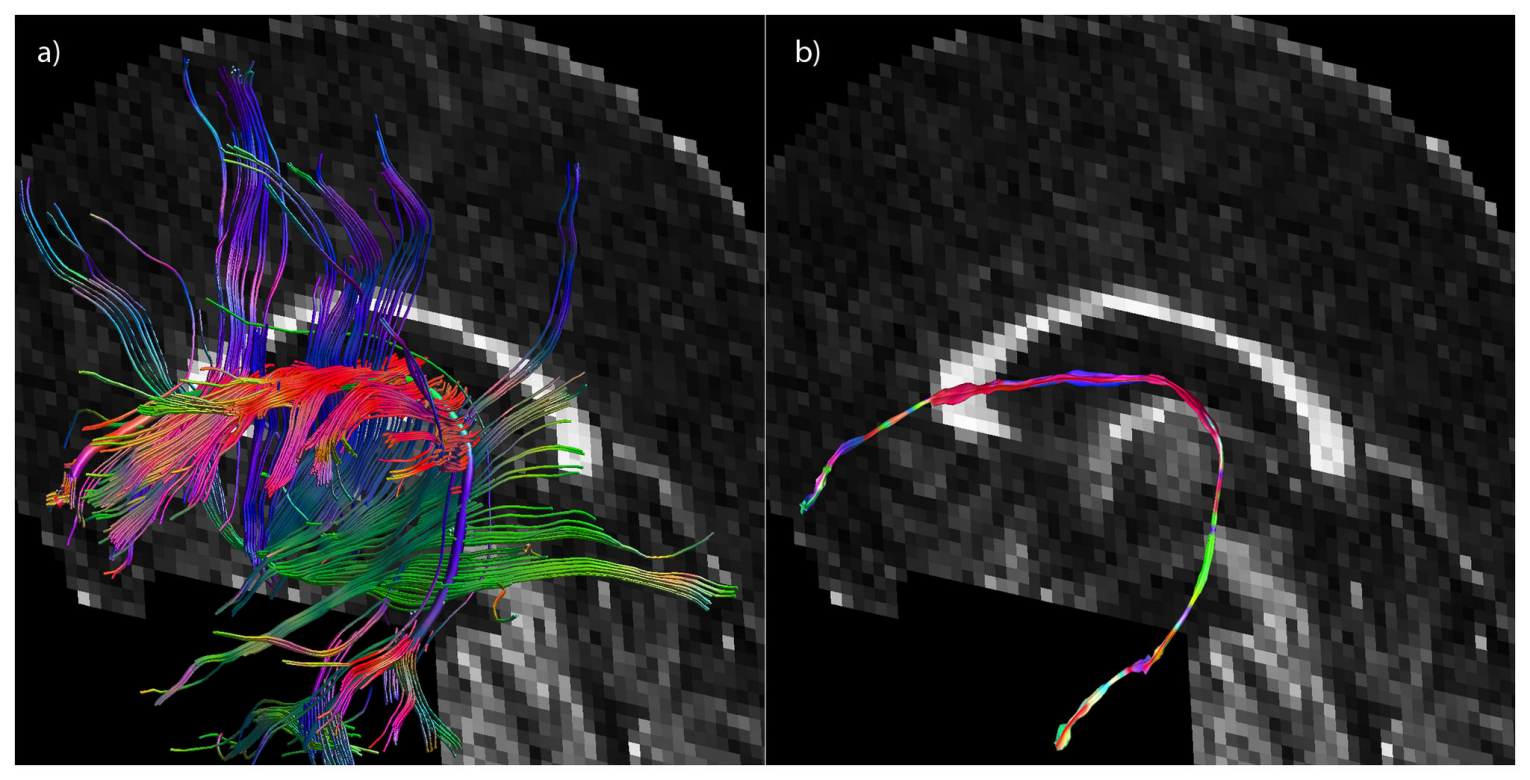
Visualization of local tract orientations crossing a fiber tract. A single tract from the arcuate fasciculus (AF), the same as in Figures 5 and 6. a) Tract shown as a thick streamtube with fibers crossing this tract of interest shown as thinner streamtubes. b) Tract shown as multi-fiber hyperstreamline.

When trying to visualize the fiber architecture along a specific fiber tract, alternatives to the multi-fiber visualization method proposed here are possible. Conceptually, it might be very helpful to show which fiber pathways cross a specific tract-of-interest, as this shows information on the fiber organization at this tract-of-interest. However, showing these perpendicularly crossing pathways can clutter the image, obscuring the view of the tract-of-interest, as shown in Figure 11a. The proposed methods provide a more succinct representation of the local tissue architecture, as shown in Figure 11b for the multi-fiber hyperstreamline. This is shown in a more detail in in Video S4 (for a single tract from the AF for a different subject).

An important and promising clinical application of fiber tractography is surgical planning. Since the initial case reports in the literature (e.g., Coenen et al. [52]), its use in surgical planning is steadily growing. A recent overview on the use of tractography has foretold a promising future, stating that it “will become established as a routine clinical investigation in many centers in the coming years” [53]. In this prospect, Ciccarelli et al. [53] believe that improvements in tractography visualizations are of paramount importance to fully benefit from tractography information. With several studies already demonstrating that post-surgical outcome is improved if fiber tracking is used in pre-operative planning [30,54], and predicting, to some degree, the clinical improvements after treatment [55,56], any improvements in visualization of fiber tracts and local tissue microstructure can only be considered beneficial for surgical outcome.

Recent research shows that the vast majority of WM voxels contains more than one fiber population with approximately 25 to 50% containing more than two fiber populations, depending on the specific diffusion model [23]. This makes the use of ODF-based methods less of a luxury and more a necessity, both in biomedical research and clinical applications (e.g., 31,57-60). More specifically, ODF-based multi-fiber tractography is beginning to show improved clinical results over tensor-based tracking, in the presence of tumors [56] and in surgical target localization for deep brain stimulation [57], which further stresses the need for multi-fiber visualization approaches.

In conclusion, we have shown that multi-fiber tractography visualizations can display the local fiber architecture along fiber trajectories, combining the detailed tissue characterization captured in the ODF with the global anatomical information from multi-fiber tractography. These new visualization approaches create a more complete picture of the WM microstructure for tracts that traverse through areas of complex fiber configurations. By facilitating the interpretation of tractography data, these multi-fiber hyperstreamlines and streamribbons may improve our understanding of white matter characteristics.

## Supporting Information

Figure S1 **Arcuate fasciculus crossing the inferior fronto-occipital fibers.** In a), the inferior fronto-occipital fasciculus (IFOF) is shown crossing a posterior part of the arcuate fasciculus (AF). Enlarging the region where these pathways cross (b) clearly illustrates that the hyperstreamline visualizes this anterior-posterior fiber population. For detailed interpretation, the hyperstreamline is shown in c) without the IFOF. In d), the hyperstreamline is colored by the number of unique fiber populations – where red indicates 1, green is 2, and blue more than 2 fiber populations.
(TIF)

Figure S2 **Conceptual illustration of the creation of a hyperstreamline.** For a point **r** along the tract, a number of points is defined in a circle around **r** perpendicular to the tract orientation **t** (a), and vectors from r to these points are calculated (b). These vectors are then scaled according to the ODF amplitude along these vectors (c), delineating the cross-sectional shape of the hyperstreamline at point **r**.
(TIF)

Video S1 **Creation of a hyperstreamline for a tract from the lateral projections of the corpus callosum.**
(MPG)

Video S2 **Creation of a multi-fiber hyperstreamline for a fiber tract from the arcuate fasciculus.**
(MPG)

Video S3 **The cortico-spinal tracts visualized as streamtubes with different color-encoding.** On the left, the conventional color-encoding was used; the ODF color-encoding was used on the right. The ODF color-encoded streamtubes show the presence, location, and orientation of crossing fiber populations along the CST, displaying only a single fiber population in the region of the internal capsule.
(MPG)

Video S4 **Visualization of local tract orientations crossing a fiber tract.** Visualization of all fiber tracts crossing such a fiber pathway can be informative to show the large-scale connectivity, but obscures the pathway of interest. By instead visualizing the local microstructure captured in the ODF, it becomes possible to visualize the complex tissue architecture locally along a fiber tract in a compact and elegant manner.
(MPG)

Appendix S1 **Pseudo-code for generating multi-fiber hyperstreamlines.**
(DOCX)

Appendix S2 **Pseudo-code for generating multi-fiber streamribbons.**
(DOCX)

Appendix S3 **Background on computational cost of visualizations.**
(DOCX)
