# Supplementary figures and images for: Multi-Fiber Tractography Visualizations for Diffusion MRI Data
Source: PLoS One. 2013 Nov 25;8(11):e81453. doi: 10.1371/journal.pone.0081453 (PMC3839966; doi:10.1371/journal.pone.0081453)

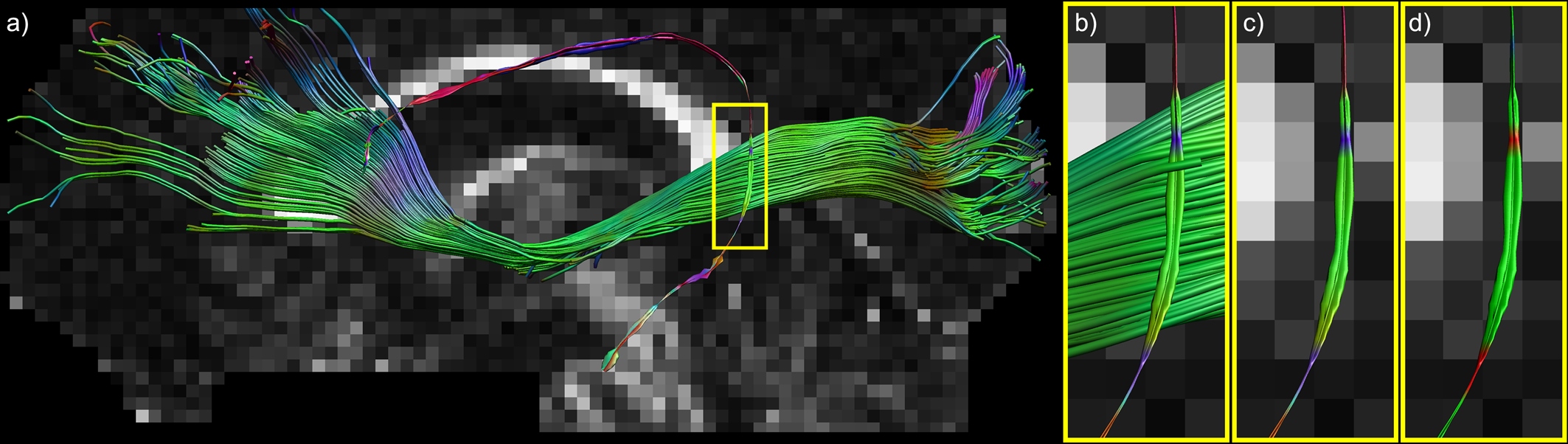

Supplement: Figure S1 — Arcuate fasciculus crossing the inferior fronto-occipital fibers. In a), the inferior fronto-occipital fasciculus (IFOF) is shown crossing a posterior part of the arcuate fasciculus (AF). Enlarging the region where these pathways cross (b) clearly illustrates that the hyperstreamline visualizes this anterior-posterior fiber population. For detailed interpretation, the hyperstreamline is shown in c) without the IFOF. In d), the hyperstreamline is colored by the number of unique fiber populations – where red indicates 1, green is 2, and blue more than 2 fiber populations. (TIF) [file pone.0081453.s001.tif]

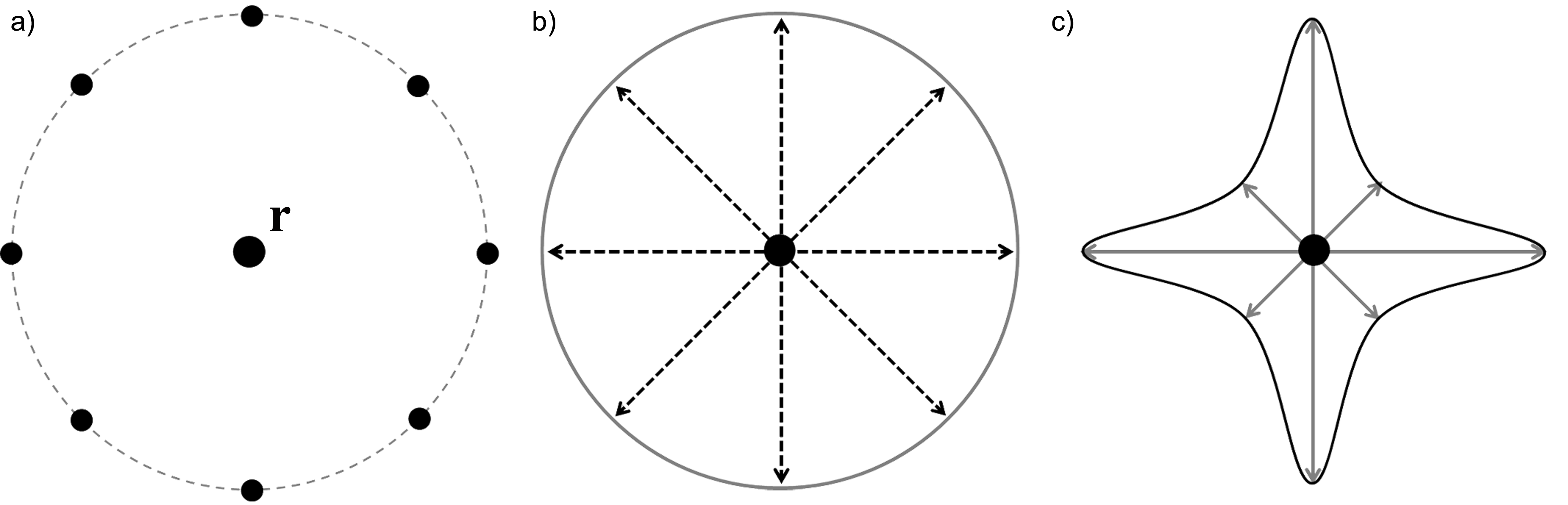

Supplement: Figure S2 — Conceptual illustration of the creation of a hyperstreamline. For a point r along the tract, a number of points is defined in a circle around r perpendicular to the tract orientation t (a), and vectors from r to these points are calculated (b). These vectors are then scaled according to the ODF amplitude along these vectors (c), delineating the cross-sectional shape of the hyperstreamline at point r. (TIF) [file pone.0081453.s002.tif]
