# Supplementary material for: Multi-Fiber Tractography Visualizations for Diffusion MRI Data
Source: PLoS One. 2013 Nov 25;8(11):e81453. doi: 10.1371/journal.pone.0081453 (PMC3839966; doi:10.1371/journal.pone.0081453)
Supplement: Appendix S1 — Pseudo-code for generating multi-fiber hyperstreamlines. (DOCX) [file pone.0081453.s007.docx]

# Appendix A1

This appendix presents pseudo-code to generate the multi-fiber hyperstreamlines.

*Calculate tract orientations,* ***t****, at each tract position,* ***r***

*Define N_crf as the number of points on the circumference of the hyperstreamline, which will define its cross-sectional area at each* ***r****.*

*for n = 1:number of tracts*

*for i = 1: number of positions along tract n*

*Define N_crf points creating a unit circle around* ***r****(n,i), in the plane perpendicular to* ***t****(n,i). % The first and last point is the same (Suppl. Fig. 2a).*

*Calculate the vector from* ***r****(n,i) to these points % (Suppl. Fig. 2b).*

*Scale the amplitude of these vectors according to the ODF amplitude at* ***r****(n,i) along these vectors % (Suppl. Fig. 2c).*

*end*

*end*
