# Supplementary material for: Multi-Fiber Tractography Visualizations for Diffusion MRI Data
Source: PLoS One. 2013 Nov 25;8(11):e81453. doi: 10.1371/journal.pone.0081453 (PMC3839966; doi:10.1371/journal.pone.0081453)
Supplement: Appendix S2 — Pseudo-code for generating multi-fiber streamribbons. (DOCX) [file pone.0081453.s008.docx]

# Appendix A2

This appendix presents pseudo-code to generate the multi-fiber streamribbons.

*Calculate tract orientations,* ***t****, at each tract position,* ***r****.*

*for n = 1:number of tracts*

*for i = 1: number of points along tract n*

*Calculate orientations and magnitudes,* ***P****, of the unique ODF peaks* ***%*** *in this work limited to 3*

*Remove the ODF peaks corresponding with the tract orientations* ***%*** *(Fig. 2f, top)*

*for a = 1:number of remaining ODF peaks*

*Create a vector,* ***K****(i,a), from* ***r****(n,i) ­–* ***P****(a) to* ***r****(n,i) +* ***P****(a)*

*end*

*Connect vectors from* ***K****(i-1) to the vectors from* ***K****(i) that have the smallest angular deviations % (Fig. 2f), In case of no peaks, the ribbon collapses to a streamline*

*end*

*end*
