# Supplementary material for: Multi-Fiber Tractography Visualizations for Diffusion MRI Data
Source: PLoS One. 2013 Nov 25;8(11):e81453. doi: 10.1371/journal.pone.0081453 (PMC3839966; doi:10.1371/journal.pone.0081453)
Supplement: Appendix S3 — Background on computational cost of visualizations. (DOCX) [file pone.0081453.s009.docx]

# Appendix A3

This appendix describes the computational cost of rendering these multi-fiber hyperstreamlines and streamribbons. All calculations have been performed on a desktop computer with a 3.40 GHz CPU, using only a single CPU for ODF estimation, fiber tractography, and calculations of the visualizations, and no dedicated GPU is required. The visualization and rendering was done using unoptimized Matlab code.

The number and orientations of ODF peaks at each point along the fiber tract – required for the visualizations – are calculated in ODF estimation and tractography, and are thus known a priori.

Calculation of the hyperstreamlines takes just under a second per hyperstreamline. When multiple tracts are to be calculated, the rendering can be parallelized over multiple CPUs. For instance, for the 87 fiber tracts of the arcuate fasciculus shown in Fig. 7b, creation of the hyperstreamlines is parallelized using 4 CPUs, which results in a computation time of roughly 20 seconds.

Calculation of the streamribbons is less expensive, and takes under a second for the 87 fiber tracts of the arcuate fasciculus (on a single CPU).
